# CLRM-04. PHASE I/II SAFETY AND EFFICACY STUDY OF BET BROMODOMAIN INHIBITOR OTX-015 WITH OLAPARIB AND LOMUSTINE IN PATIENTS WITH RECURRENT GLIOBLASTOMA

**DOI:** 10.1093/noajnl/vdab112.003

**Published:** 2021-09-21

**Authors:** Fred Lam

**Affiliations:** 1 Northwell Health, Manhasset, NY, USA; 2 Koch Institute for Integrative Cancer Research at MIT, Cambridge, MA, USA

## Abstract

Standard of care for patients with glioblastoma (GBM) includes resection with concurrent temozolomide (TMZ) and radiotherapy, with inevitable disease recurrence. Upon recurrence, tumors are often resistant to first-line therapies and/or have infiltrated eloquent or deep brain regions, precluding repeat resection. There is currently no standard of care for recurrent GBM and patients succumb to their disease burden within 12- 15 months of their initial diagnosis of recurrence, exposing an unmet need to find novel therapies to treat recurrent disease. Bromodomain and extraterminal (BET) proteins are chromatin readers that affect transcription of genes. The oral BET inhibitor (BETi) OTX-015 has shown promise in a dose-escalation, phase I study in patients with acute leukemia and other BET inhibitors are currently in phase I studies for the treatment of primary brain tumors. We have recently shown that BET inhibition increases DNA damage and mitotic catastrophe in oncogenic cells by increasing transcription-replication conflicts and downregulating expression of key DNA damage checkpoint proteins, and have also shown its efficacy in decreasing tumor burden and improving survival when combined with TMZ in intracranial mouse models of glioma. We have also demonstrated that BETi's synergize with Olaparib by downregulating expression of the BRCA-driven DNA damage repair pathway and further leverages additive effects when triply combined with other DNA damaging agents such as Lomustine to decrease tumor burden and improve survival in patient-derived mouse models of GBM and medulloblastoma. We therefore hypothesize that the synergistic and additive effects of this triple combination seen in our preclinical studies will achieve therapeutic benefits in patients with recurrent GBM.

